# Family Planning in the Republic of Tajikistan: A narrative review from Soviet times to 2017

**DOI:** 10.5195/cajgh.2020.404

**Published:** 2020-03-31

**Authors:** Ellison Henry, Zarrina H. Juraqulova

**Affiliations:** 1 Maternal and Child Health Department, Gillings School of Public Health, University of North Carolina at Chapel Hill, Chapel Hill, North Carolina, USA; 2 Economics Department, Denison University, Granville, Ohio, USA

**Keywords:** Tajikistan, Family planning, Fertility, Family size, Soviet, Women

## Abstract

**Introduction:**

Tajikistan's dramatic shift from a high to a low fertility society has taken place over a little more than two decades. While some fertility beliefs remained the same throughout the rapid economic and political transitions of Tajikistan, other beliefs may have changed to respond to the financial realities of the newly independent and Central Asian republic, such as having fewer children. The objective of this review was to describe and analyze the state of family planning in the Republic of Tajikistan from Soviet period (1929-1991) until 2017.

**Methods:**

The review is based on materials obtained from various sources including Google Scholar and PubMed, relevant to family planning in Tajikistan, including government policies, open-access nationally representative data, journal articles, and program reports, identified through a selective search of Google Scholar and PubMed databases, and the grey literature.

**Conclusions:**

This narrative review presents the history of family planning in Tajikistan, outlines an understanding of the health system context as it relates to family planning, and analyzes the latest national family planning policy (2017). The authors suggest further research is required to (a) understand the beliefs and practices related to family planning; and (b) define strategies to address the issue of unmet need of family planning services.

During the Tajik Soviet Socialist Republic's (Tajik SSR) membership within the Soviet Union (USSR) from 1929–1991, pro-natalist policies were in place. USSR aimed to expand the population of member republics to meet economic targets defined in five-year plans. In that period, it was possible to support high fertility rates with financial and other resources provided by the centralized government in Moscow. Cheap housing, free education and health care, free plots of land for members of communal and state farms, and the low costs of essential food stuffs all made it possible for most families to afford the economic costs of many children without too much hardship.^1^ The Tajik SSR, along with today's Central Asian republics, had the highest fertility rates within the USSR. According to census data, the Tajik SSR experienced the highest average annual rates of population growth between 1959–1970, 1970–79, and 1979–1989.^2^

The Soviet health system was designed to provide uniform quality of services across member republics; however, there were large variations in the number of health workers and the range and quality of services provided. For example, in 1987, Georgia (5.7) had more than two times the number of physicians per 1,000 population in comparison to Tajikistan (2.7). Per international agreements, the government supported family planning service provision and utilization, though in practice, it was not promoted.^3^^–^^6^ After legalization in 1955, abortion was the primary method of fertility control throughout the USSR.^3^,^5^,^7^ The Ministry of Public Health published abortion statistics in 1988, the first such publication in nearly 60 years. This report confirmed the use of abortion as the primary method of contraception and reported wide use of traditional methods of contraception (i.e. withdraw, rhythm) among those who used any method. In 1988 there were nearly 5.8 million abortions in the USSR.^3^ Shortages of modern contraceptives coupled with an easily accessible network of abortion clinics and lack of access to family planning education were primary contributors to low utilization of modern methods.^3^^–^5

In 1989, as a response to people's growing concerns about abortion safety, the Soviet Family Health Association was created. This NGO focused its efforts on family planning initiatives. Further, in 1990 the government created the Committee on Problems of Women, Family and Maternal and Child Health Care.^5^

Researchers have been cautious in interpreting data from the USSR due to possible lack of completeness and questions of validity and reliability.^3^,^7^ Available data on contraceptive method mix is largely based on behaviors of women in Moscow. Surveys conducted between the 1960s–1980s report a consistent trend of preference for traditional methods–withdraw and rhythm–followed by condoms.^3^ Researchers used 1990 survey data to estimate fertility rates and contraceptive prevalence rates across member republics. According to these estimates, the Tajik SSR's high fertility rate was paired with the lowest contraceptive prevalence rate; only three percent of married couples reported using any method of contraception.^7^ The purpose of this narrative review of the literature is to describe and analyze the state of family planning in the Republic of Tajikistan from Soviet time until 2017. This review is the first to synthesize family planning literature from academic and non-academic sources within the context of Tajikistan using the most updated nationally representative survey, the 2017 Demographic and Health Surveys (DHS).

## Methods

A narrative review of English articles was conducted using two databases (Google Scholar and PubMed), grey literature and reference lists of key articles. All search results were reviewed and included if they addressed the key outcome of family planning within the context of Tajikistan. Secondary searches were conducted to include articles addressing fertility and/or family planning in the USSR and Central Asia. Articles were included if data presented were disaggregated to republic-level statistics.

“Grey literature” refers to publications outside of academia and peer-reviewed journals that are disseminated by governments and organizations. The authors searched grey literature to identify relevant government policies and reports from nonprofits and donor agencies. These documents were included if directly pertaining to family planning in Tajikistan.

The authors conducted a search for open-access nationally representative data. After identifying several such surveys (i.e. MICS, TLSS, DHS), the authors chose to include the 2012 and 2017 DHS as primary data sources for this review. The DHS were chosen because they provided the most thorough and updated data on family planning. The literature as it relates to family planning in Tajikistan is centered on other primary outcome measures, using family planning as a dependent variable.^^8^^-^^10^^ The authors offer this review as a contribution to the family planning literature, as a starting point to understanding the current status of family planning in Tajikistan and how the country's related public health efforts have developed since independence.

By focusing on English-language publications with a focus on Tajikistan, it is possible that the authors missed seminal publications that otherwise met the inclusion criteria but were published in Russian language or focused on other Central Asian Republics. The authors chose to limit their scope within these broader language and geographical parameters.

## Discussion

### Women, Marriage and Fertility

Tajikistan is officially a secular state; however, since independence there has been a resurgence of some traditional Islamic (Tajik) values that define a woman's main role in the private sphere (i.e. in the home). The government has publicly promoted the traditional interpretation of the role of women in Tajik society.^11^,^12^ As Roche notes, “&post-Soviet domestic gender politics have focused primarily on women in their role as glorified mothers. Along with a revival of Islam since independence, motherhood has been reinforced as a sacred status by both the government and the Islamic opposition, albeit in different ways.”^13^ The effect this may have on women's roles in Tajik society has not been thoroughly studied.^14^,^15^

There are rather significant generational differences among women in Tajik society. For example, today's young women navigate a space that is somewhere between progressive (i.e. Soviet) and traditional (i.e. Tajik). Soviet policy and propaganda promoted gender equality and the participation of women in the public sphere. The authors note certain aspects of Soviet life may have reinforced traditional gender roles, particularly regarding women's reproductive lives (i.e. mother awards and birth incentives). The traditional patriarchal influences of Tajik culture promote the role of women as mothers and homemakers, i.e. as a central figure in the private sphere.^14^,^15^

The empowerment of women has broad implications for improving health outcomes and vice versa. The ability to control fertility is associated with a woman's status in the home and her sense of self-worth. Both the 2012 and 2017 DHS examined the degree of women's empowerment among married Tajik women and its relationship with family planning decisions. Both DHS reported that contraceptive use was “…positively associated with women's participation in household decision-making.” The surveys asked women about three types of decisions: those related to their own health care, major household purchases, and visits to their family or relatives.^16^,^17^ Using the 2012 DHS, Juraqulova and Henry (2020) examined the impact of decision-making abilities on women's contraceptive behavior. The authors found that the probability of using birth control was higher for a woman who reported having voice in household decisions *and* the financial means to get medical help, compared with women who do not have both or either of these choices.^18^

The shock of political and economic transition from being a member of the USSR to an independent republic prompted families, especially women of reproductive age, to adopt family planning to achieve desired family size. In the “new” economy, one dependent on household-generated resources to establish and maintain a level of well-being, couples who once had ten or more children during the Soviet period now have four or fewer.^16^,^17^ Tajikistan navigated three socioeconomic challenges that contributed to a national reduction in family size: civil war (1992–1997); food crisis (1995); and drought (2000–2001), which caused food shortages. A fourth factor contributing to the fertility decline was a temporary labor migration of working-aged males.^19^ Labor migration continues to impact marriage and childbearing.^20^,^21^

**Figure 1.**
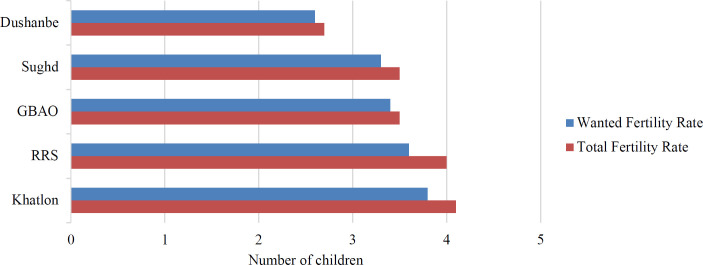
Wanted and total fertility rates, Tajikistan, 2017

Marriage is a central event in Tajik culture, especially in rural areas. It is often the woman's first exposure to sexual activity and the possibility of pregnancy.^1^ As of 2017, about 75% of Tajik women aged 15–49 reported being currently married.^17^ Societal expectations necessitate newlyweds have a child within one year of union. Approximately seven percent of teenage girls aged 15–19 in Tajikistan have begun childbearing.^17^ Figure 1 shows that women in every oblast report having more children than desired. The difference between wanted and actual fertility rates suggest an unmet need for family planning services throughout the four oblasts and the capital, Dushanbe.^16^,^17^ An unmet need for family planning occurs when women are unable to space and/or limit childbearing as they desire.^22^

### Family Planning

The Ministry of Health and Social Protection of the Population (MoHSPP) prioritizes family planning under the umbrella of reproductive health and oversees providing family planning guidance to local-level implementers (i.e. oblast-level facilities). The Ministry's main objectives, specific to family planning, include:

1. “Ensuring low-risk pregnancy and safe motherhood; and
2. Reducing complications caused by closely-spaced pregnancies and pathological conditions among women of reproductive age.”^23^

The Ministry is responsible for training providers, providing “extensive” family planning education to all Tajiks, and supplying contraceptives throughout the nation. Family planning services are provided at regional, district and city reproductive health centers at the primary health center level and monitored by the National Center on Reproductive Health.^23^ These departments work in conjunction with local city authorities to define and deliver health services throughout facilities in each oblast.^6^

In the Strategic Plan for Reproductive Health (2005–2014) the MoHSPP formulated an initiative to improve the reproductive health of Tajiks, specifically women, by:

1. Increasing access to family planning services and contraceptives;
2. Increasing access to antenatal and safe delivery services; and
3. Decreasing mortality and morbidity during pregnancy and improving perinatal outcomes.^6^

While the objective of the plan was to integrate reproductive health services into primary care services, the program has yet to be successfully defined and implemented. Consequently, measures to increase public awareness of the importance of reproductive health and to train adequate human resources to meet reproductive health needs have not been fully implemented.^6^

The most recent reproductive health and family planning implementation policy, passed in 2017, recommends key interventions to: (a) create an enabling environment favorable to reproductive health and family planning; (b) adapt the health system to address reproductive health challenges; and (c) provide programs that meet the needs of adolescents and youth.^24^

The distribution of functioning health facilities, funding for health services and provision of care is uneven across Tajikistan. The overall ratio of health worker to population has declined over the last twenty-five years. Today, there are fewer health professionals per capita in Tajikistan than any other country in Central Asia. Outmigration of educated and skilled medical providers has been a significant contributing factor. Health worker shortages are most severe in rural areas. This is the result of low salaries, outdated medical equipment, poor human resource training, re-training and management, and the deteriorating health infrastructure.^6^ While the MoHSPP advocates for a ratio of one family medicine physician per 1,500 population and one family nurse per 750 population across the country, most physicians and nurses are concentrated in the capital, Dushanbe. Currently, the combined ratio of physicians and nurses to population is one of the lowest in the WHO European Region.^6^

Mid-level medical professionals typically practice in rural areas, carry out preventive and diagnostic tasks and perform some administrative duties. Nurses train for four years but hold low status within the medical community and extremely modest salaries when compared with physicians.^6^ Assumptions could be made about which level of medical provider is responsible for providing family planning services and reproductive health counseling, but specific responsibilities remain unclear and are not specified in the national Implementation Plan for Family Planning Services (2017–2020).

The majority of Tajikistan's health expenditures come from out-of-pocket payments for services, primarily payments for curative care. In 2014 a total of US$1.7 billion was spent on curative health care services, over half of which came from out-of-pocket payments.^25^ It is estimated that by the year 2040, around 40% of all funds spent on health will come from out-of-pocket payments.^26^ While the government promotes family medicine and preventative programs, government (oblast-level, i.e. *hukumat*) financing gives primary attention to hospital services and continues to be dominated by input-based budgeting that is based on the number of hospital beds and/or personnel rather than on health objectives, quality of care and outcomes.^6^,^27^

**Figure 2:**
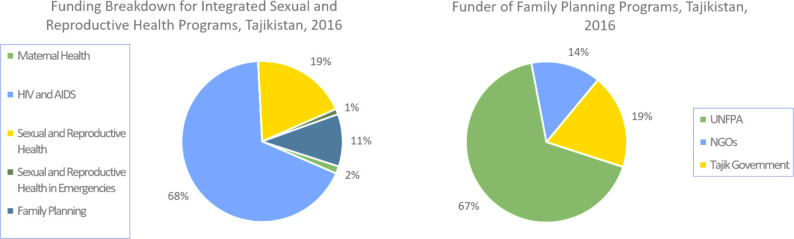
Family planning program funding, Tajikistan, 2016

The United Nations Population Fund (UNFPA) has been the largest donor of reproductive health and family planning services in Tajikistan. In total, 40% of all integrated sexual and reproductive health programs in 2016 were financed by UNFPA; other NGOs were responsible for 54% of the financing. The Tajik government financed less than 5% of integrated reproductive health programs (Figure 2, right side).^28^ Figure 2 (left side) shows 2016 data, which reported about US$130,000 (11%) of the total integrated sexual and reproductive health service funding was set aside to finance family planning initiatives. UNFPA funded 67% of the family planning-specific programs in 2016.

**Figure 3.**
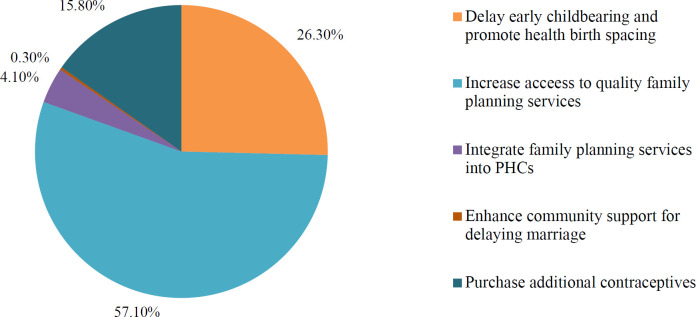
Distribution of funds by priority action area, Costed Implementation Plan for Family Planning, 2017-2020

The 2017–2020 national policy on family planning includes the MoHSPP's estimate of the total funding needed for family planning and proposal for how those funds will be allocated. However, the plan does not designate the source(s) of funds nor indicate how the projected program costs were calculated. Figure 3 shows the distribution of funds by action area; in total these efforts are estimated to cost about US$1.9 million.^24^ Around US$1.6 million will be spent on interventions, the remaining funds are allocated to purchase contraceptives required to meet the target contraceptive prevalence rate increase of 1.5% per year for each of the plan's years (2017–2020). The source for these contraceptive methods and a plan for distribution and use were not identified. Within the US$1.6 million for programs, about US$110,000 is slated for provider education. US$964,000 is allotted to “increase access to family planning services and methods”. This increase will be achieved through additional provider training and a bit of service monitoring in selected facilities.^24^ There is no mention of efforts to train *additional* providers, retain those graduating from medical education programs or to extend family planning services more equitably across the country.

According to the 2012 and 2017 DHS, most Tajik women received family planning information via a home visit by a health worker or a facility-based provider. Women in Khatlon (16.1%) were less likely to be visited in the home than women in other regions in 2012. However, in 2017 home visits by health workers increased for women in Khatlon by 11.2%, perhaps a consequence of USAID's Feed the Future program activities that promote home health visits. Women in Dushanbe experienced the greatest percentage increase of home visits, from 18.6% in 2012 to 32.1% in 2017.^16^,^17^

It is unclear how women in both Khatlon Oblast and Regions of Republican Subordination receive reproductive health information if they lack access to a home health visitor or a facility-based provider. Fifty three percent of women had not received any messaging from radio, TV or print media (i.e. newspaper, magazine).^17^ Many women may be subject to myths about family planning and reproductive health and are unaware of useful family planning information.

The 2017 DHS reported about 71% of currently married women were not using any method of contraception (modern or traditional) as compared with 72% reported in the 2012 DHS. The top three reasons

women reported discontinuation of use were wanting to become pregnant (32.6%), side effects/health concerns (26.3%) and infrequent sex/husband away (17.7%). Eighteen percent of women using any method of contraception reported using the intrauterine device (IUD), and a marginal percentage used pills, male condoms, injectables or traditional methods. In total, 20% of women want to stop having children while 11% want another child in two or more years.^17^ This suggests that 33% of married women in Tajikistan need access to long acting contraceptive methods (i.e. implant or sterilization) to achieve fertility goals.

The DHS identified an association between decision-making power and unmet need for family planning. The use of *any* method of contraception and any *modern* method both steadily increased with the number of household decisions women participated in (i.e. those related to their own health care, major household purchases, and visits to their family or relatives). Tajik women who reported not participating in household decisions had the highest unmet need (25%), while those who participated in all three measured decisions had lower unmet need (21%) according to 2012 DHS. However, these numbers changed in 2017 – unmet need was higher among women who participated in all three decisions (34.6%) compared to women not taking part in any decisions (23.7%).^17^

In the Costed Implementation Plan for Family Planning (2017-2020), the MoHSPP identified unmet need of family planning as a specific area of public health concern. The government proposed a target of 1.5% annual increase in the contraceptive prevalence rate (CPR) as a pathway to reducing unmet need.^24^ However, the plan does not provide the details of *how* CPR will be increased; it does indicate that “to reach this goal a number of key interventions were developed making use of the…expertise of national experts and technical assistance partners”.^24^

## Conclusion

In 2014, with support from USAID, EngenderHealth's RESPOND project used DHS data to work with the MoHSPP and other national stakeholders to project fertility and contraceptive prevalence trends in Tajikistan until 2020. RESPOND published a report specifying the inputs (i.e. finances, contraceptive methods) necessary to achieve two outcomes of interest: (1) maintain current CPR among married women of reproductive age through 2020; and (2) reduce the current unmet need by 2020 by providing a more balanced method mix. An estimate of US$1.3 million was given to meet the first outcome, and an additional US$450,000 to achieve the second outcome. The source of funds and other recommended inputs were not identified.^29^

The RESPOND activity and most recent national implementation strategy are encouraging steps towards providing the reproductive healthcare services that Tajik women seek to achieve fertility goals. The 2017-2020 Costed Implementation Plan for Family Planning suggests reproductive health is a core component of the political agenda of the Tajik government.

This narrative review has collected relevant information about family planning in Tajikistan, from Soviet time until 2017. Data has been provided to show that women experience an unmet need for family planning and access to these services is insufficient for women to achieve desired fertility. Research is needed to provide an understanding of the family planning beliefs and practices of key populations. For example the beliefs and practices of women in Khatlon Oblast, an important location because both DHS surveys reported Khatlon's unmet need and fertility rates as the highest in Tajikistan, coupled with early marriages and low contraceptive method uptake.^16,17^ Further research is crucial to develop an in-depth understanding of women and provider experiences utilizing and providing family planning services. This information is necessary to inform strategies to address unmet need and inequitable access to quality services across the country. Reproductive health and family planning relate to all aspects of population health and must be maintained as pillars of Tajikistan's future health policies and reforms.
